# Nuclear Factor Kappa B, Matrix Metalloproteinase-1, p53, and Ki-67 Expressions in the Primary Tumors and the Lymph Node Metastases of Colorectal Cancer Cases

**DOI:** 10.1155/2015/945392

**Published:** 2015-04-06

**Authors:** Ibrahim Meteoglu, Ibrahim Halil Erdogdu, Pars Tuncyurek, Adil Coskun, Nil Culhaci, Muhan Erkus, Sabri Barutca

**Affiliations:** ^1^Department of Pathology, Medical Faculty, Adnan Menderes University, 09100 Aydin, Turkey; ^2^Department of Pathology, Medical Faculty, Adıyaman University, 02100 Adiyaman, Turkey; ^3^Department of General Surgery, Medical Faculty, Adnan Menderes University, 09100 Aydin, Turkey; ^4^Department of Gastroenterology, Medical Faculty, Adnan Menderes University, 09100 Aydin, Turkey; ^5^Department of Medical Oncology, Medical Faculty, Adnan Menderes University, 09100 Aydin, Turkey

## Abstract

Colorectal cancer (CRC) is the third most frequent malignancy. Many factors such as NF-*κ*B, matrix metalloproteinase-1 (MMP-1), p53, and Ki-67 are likely to be involved in its development and progression. Lymph node metastases indicate increased tumor burden and tumor cell heterogeneity and affect both the treatment strategies and the prognosis. In this study, expressions of NF-*κ*B, MMP-1, p53, and Ki-67 were between the primary tumors and lymph node metastases in 110 Dukes' stage C, CRC cases by immunohistochemical methods, related to patients' clinical outcomes. NF-*κ*B, p53, and Ki-67 expressions were significantly higher in the metastatic lymph nodes compared to the primary tumor tissues (*P* = 0.04, *P* = 0.04, and *P* = 0.01, resp.). In the metastatic lymph nodes NF-*κ*B expression was correlated with both p53 (*r* = 0.546, *P* = 0.003) and Ki-67 (*r* = 0.586, *P* = 0.0001) expressions. The univariant and multivariant analyses showed that only “pT stage” preserved an independent prognostic significance for recurrence-free survival rates and 5-year overall survival rates (*P* < 0.001 for both). Metastatic cells can acquire different biological characteristics compared to their primaries. Elucidation of properties acquired by metastatic cells is important in order to better determine prognosis, reverse drug resistance, and discover new treatment alternatives.

## 1. Introduction

Colorectal cancer (CRC) is one of the most common malignancies that has high morbidity and mortality throughout the World, especially in developed countries [1, 2]. Existence of lymph node metastasis is an important prognostic indicator in CRC. The rate of long-term survival is decreased dramatically in cases with Dukes' stage C disease that show lymph node metastases compared to earlier stages. Metastatic tumor cells can acquire different phenotypical and biological characteristics compared to their primaries [3]. Most of the operable tumors have regional lymph node metastases so it is important to determine the variations in the biological characteristics of the metastatic cells, especially to develop a better strategy for adjuvant treatment modalities [4]. Recently, the evaluation of biomarkers to assess treatment alternatives for CRC has become popular for researchers and pathologists, and the clinical importance of cell cycle changes and gene alterations has evoked great interest in research from pathological points of view [5, 6].

The variations in the biological characteristics of tumor cells in the metastatic tissues have an influence on their ability to survive [7]. A variety of factors, including apoptosis, proliferation potential, and genetic alterations, have regulatory roles in the life-cycle of spreading tumor cells [7–9].

NF-*κ*B signaling is of importance to cell proliferation, apoptosis, and creation of the inflammatory response and has been found to be implicated in the development of many cancers [10]. There have been studies showing that NF-*κ*B affects CRC development and especially carcinogenesis after chronic inflammations. Several studies have revealed that NF-*κ*B can be used as a marker of resistance to adjuvant chemotherapy in CRC [11–14].

There is also evidence that matrix metalloproteinases (MMPs) might be a modulator of tumor progression and spread, which affect the prognosis of the disease [15–17]. In addition, growing evidence indicates that MMP has a role in tumor progression and spread, apart from its role in tissue injury [18, 19]. Many studies suggested that MMPs may also have some roles in CRC biology. It has been emphasized that MMP-1 (interstitial collagenase) expressions are important in the prognosis and the progression of CRC [20–22]. However, the role of MMP in the tumor cells of the metastatic lymph nodes has not been elucidated. There have been few studies on the potential relationship between NF-*κ*B and MMP. NF-*κ*B activation has been reported to be involved in the regulation of some MMP types [23, 24].

It has been shown that mutations of p53, as a well-defined tumor suppressor gene, contribute to the development of many cancer types and that p53 is a poor prognostic factor in many cancer types [24–30]. It was previously reported that its expression in primary tissues and metastatic axillary lymph nodes did not differ in breast cancer cases [3]. However, p53 may associate with lymph node metastases and poor prognosis in CRC.

Ki-67 is a proliferation marker, observed in all phases of cell cycle except G0. High Ki-67 expression is a poor prognostic factor in many tumors and there are correlations between Ki-67 expression and expression of other poor prognostic factors. In a previous study, Ki-67 expression was found to be higher in metastatic lymph node tissues compared to primary in breast cancer [3]. In CRC, high proliferation rate can be related to tumor size, metastases, and differentiation. Nevertheless, it is still unclear whether there is a correlation between altered NF-*κ*B, MMP, p53, and Ki-67 production and the clinical behavior of metastatic tumor cells.

A series of unique biological features may affect the ability of cancer cells to create metastasis. It is important to reveal these biological characteristics of metastatic cells in CRC in terms of development of new therapeutic strategies. However, to our knowledge, studies are needed on the variations of expression patterns of these markers in the metastatic lymph nodes in CRC cases. Therefore, in this study, NF-*κ*B, MMP-1, p53, and Ki-67 expressions in the primary tumor tissues and metastatic lymph nodes were compared in Dukes' stage C, CRC cases.

## 2. Patients and Methods

A total of 457 CRC patients were operated on in Adnan Menderes University Hospital, Aydin, Turkey, between August 1998 and December 2010. Of these, 152 with stage Dukes' C were evaluated in this study; however, 42 were excluded as either their paraffin blocks were not available for pathologic review and/or their clinical follow-up data were incomplete. None of the patients had received chemotherapy and/or radiotherapy in the preoperative period. There was no history of previous cancer, familial polyposis coli, or preexisting inflammatory bowel diseases. Preoperative evaluations, based on clinical examination, complete blood count, blood biochemistry, chest X-ray and/or computed tomography (CT), abdominal ultrasonography and/or CT, and bone scintigraphy did not show distant metastasis in any of the patients. Ethical approval was obtained from the ethical committee of the university (ADUBAPTPF-12.0141). Tissue samples from both the primary tumors and the metastatic lymph nodes were obtained in all 110 patients. The selected samples did not include the areas of necrosis, hemorrhage, inflammation, or technical deformation. The samples from the primary tumors were representative of the histological grade of each tumor and the samples from the lymph nodes (LN) were obtained from the largest area of metastases. Microscopic examinations of the haematoxylin-eosin- (HE-) stained slides obtained from the paraffin blocks were used to confirm the diagnoses.

### 2.1. Immunohistochemistry

All immunostaining was carried out at room temperature using DAKO Autostainer Universal Staining System (Autostainer Link 48 DAKO, Glostrup, Denmark). First, sections 4 *μ*m in thickness obtained from selected paraffin embedded blocks were put on positively charged slides. Second, all the sections were deparaffinized in xylene and dehydrated through a graded series of ethanol solution. Third, antigen retrieval was performed at 96°C (10 mM/L citrate buffer, pH 6) for 40 minutes in a thermostatic bath (PT Link). The sections were incubated with NF-*κ*B/p50 (RB-1648; NeoMarkers, Fremont, CA, USA), MMP-1 (MS-1687-R7 NeoMarkers, CA, USA), anti-Ki-67 monoclonal antibody MIB-1 (M724029, DAKO, Glostrup, Denmark), and anti-p53 (M700129, DAKO, Glostrup, Denmark) for 60 minutes at room temperature. Positive and negative controls were added for each antibody and to each batch of staining. A streptavidin-biotin enhanced immunoperoxidase technique (K8000 Envision Flex, DAKO, Glostrup, Denmark) in an automated system was used to show immunoreactions. The sections were incubated with DAB and counterstained lightly with haematoxylin to demonstrate binding. Finally, the sections were dehydrated and mounted onto the slides. The slights known to be positively immunostained were used as positive controls. Normal rabbit serum IgG was used to replace primary antibody as a negative control.

### 2.2. Evaluation of Immunostaining

Sections were reviewed by 3 pathologists (Ibrahim Meteoglu, Ibrahim Halil Erdogdu, and Nil Culhaci), who were blinded to the patients' clinicopathological characteristics and follow-up information, and discrepancies (about 12% of cases) were resolved by joint discussion of the slides viewed with a multiheaded microscope. Immunostaining was scored (as described by Zhigang and Wenlv) based on the intensity of staining and the percentage of cells that stained positively [31]. The intensity of expression was graded on a scale of 0 to 3+ with 3 being the highest expression observed (0, no staining; 1+, mildly intense; 2+, moderately intense; 3+, severely intense). The staining density was quantified as the percentage of cells stained positive for immunohistochemical antibodies, as follows: 0 = no staining; 1 = positive staining in <25% of the sample; 2 = positive staining in 25–50% of the sample; 3 = positive staining in >50% of the sample. Intensity score (0–3+) was multiplied by the density score (0–3) to yield an overall score of 0–9 for each specimen, that is 0, negative expression; 1-2, weak expression; 3–6, moderate expression; and 7–9, strong expression.

### 2.3. Statistical Analysis

All statistical analyses were performed with SPSS (Windows version 13.0, SPSS Inc., Chicago, IL, USA). The Spearman correlation test was used to determine correlations between the expression of biological markers in the primary tumor and the expression of these markers in the lymph nodes. The Chi-squared test or Fisher's exact probability test was used to compare the recurrence rates. Logistic regression analysis was conducted on the parameters found to be significantly associated with recurrence by the Chi-squared tests or Fisher's exact probability test (*P* < 0.05) to identify the independent factors of recurrence. Survival rates were calculated by the Kaplan-Meier method and compared by the log-rank test. Stepwise forward Cox regression model was conducted for parameters found to be significantly associated with survival by the log-rank test (*P* < 0.05) in order to identify the independent factors of survival. Values of *P* < 0.05 were considered significant in all analyses.

## 3. Results

Clinicopathological characteristics of the patients are summarized in Table 1. The patients were mostly male (66%) and pT categories, pT3 (65.5%) and pT4 (26.3%), were predominant. The mean number of dissected and metastatic lymph nodes was 18 ± 6 and 3 ± 1.2, respectively.

**Figure 1:**
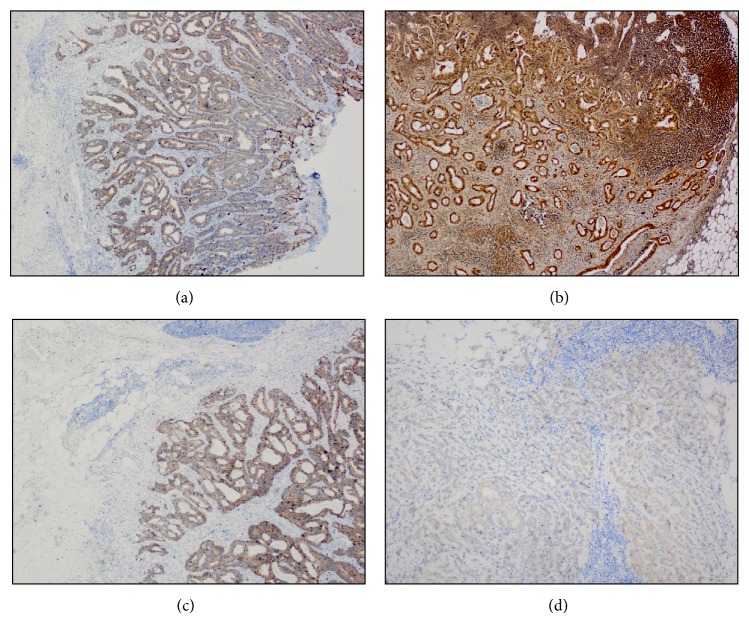
Immunohistochemical staining of (a) NF-*κ*B in a primary tumor (×40), (b) NF-*κ*B in a metastatic lymph node (×40), (c) MMP-1 in a primary tumor (×40), and (d) MMP-1 in a metastatic lymph node (×100).

**Figure 2:**
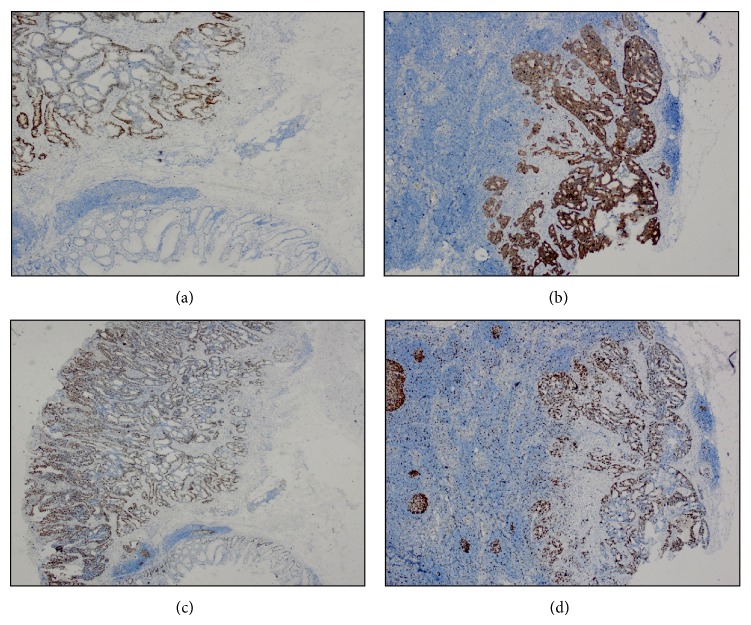
Immunohistochemical staining of (a) p53 in a primary tumor (×40), (b) p53 in a metastatic lymph node (×40), (c) Ki-67 in a primary tumor (×40), and (d) Ki-67 in a metastatic lymph node (×40).

The immunohistochemical NF-*κ*B, MMP-1, p53, and Ki-67 expressions are presented in Table 2. Examples of immunohistochemical staining of NF-*κ*B and MMP-1 in a primary tumor and a metastatic lymph node sample are shown in Figure 1 and of p53 and Ki-67 in a primary tumor and a metastatic lymph node sample are shown in Figure 2, respectively.

The rate of cells stained (moderate + strong) with NF-*κ*B, p53, and Ki-67 was significantly higher in the metastatic lymph nodes compared to the primary tumors (*P* = 0.04, *P* = 0.04, and *P* = 0.01, resp.). However, the rate of cells stained with MMP-1 was not statistically different between the primary tumor and the metastatic lymph nodes (*P* = 0.07).

NF-*κ*B expression was significantly correlated with p53 (*r* = 0.546, *P* = 0.003) and Ki-67 expression (*r* = 0.586, *P* = 0.0001) in the metastatic lymph node tissues. However, NF-*κ*B expression was not correlated with p53 (*r* = 0.215, *P* = 0.114) and Ki-67 (*r* = 0.226, *P* = 0.126) expression in the primary tumor tissues. MMP-1 expression was not correlated with any markers in the primary and the metastatic tumor tissues. Likewise, there was not a significant difference between p53 and Ki-67 expressions.

The correlation between immunohistochemical Ki-67 staining in the primary tumors and the number of metastatic nodes (*r* = 0.518, *P* = 0.018) was significant. Similarly, the size of the primary tumor was correlated with Ki-67 staining in the primary tumors (*r* = 0.328, *P* = 0.041). Furthermore, expressions of the other markers in the primary tumors and metastatic lymph nodes were not associated with any clinicopathological parameters.

**Figure 3:**
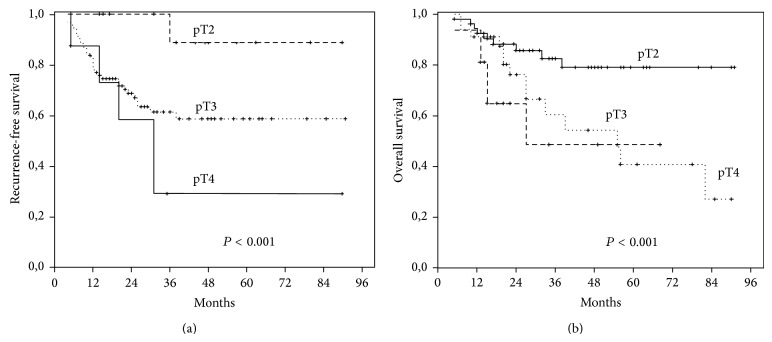
Kaplan-Meier survival analyses of pT stages. Recurrence-free survival (a) and 5-year overall survival (b) curves.

In the postoperative follow-up periods of the patients (median 26.8, min. 1.5–max. 94.4 months) the 5-year overall survival rates were 46.8%. Recurrence was observed in 42 patients (38.2%) and 68 (61.8%) had died during the follow-up. The recurrence rates in pT2, pT3, and pT4 tumors were 22.2%, 33.3%, and 55.2%, respectively. The 5-year overall survival rates in pT2, pT3, and pT4 tumors were 69.9%, 55.3%, and 32.8%, respectively. There were statistically significant correlations between the pT stage and both recurrence-free survival (RFS) (*P* = 0.01) and 5-year overall survival (OS) (*P* = 0.01) rates. No significant correlations were observed among other clinical parameters and the immunohistochemical staining scores of the studied markers. In the univariant and multivariant analyses only “pT stage” of the tumor was a significant independent prognostic factor for both RFS and 5-year OS rates (HR, 1.48; 95% CI, 1.02–2.44; *P* < 0.001 and HR, 9.782; 95% CI, 2.732–39.757; *P* < 0.001, resp.) (Figure 3).

## 4. Discussion

Although CRC is one of the most common malignancies, carcinogenesis and many factors influencing the clinical course of this disease have not been completely described. Understanding the pathogenesis and the factors affecting the development and the progression of CRC would result in better prediction of the disease course and selection of appropriate targeted treatments for patients refractory to classical chemotherapy and radiotherapy [32]. It is of particular importance to determine the biological and clinical features discriminating the metastatic tumor cells from primary tumor cells especially in advanced stages of CRC. It has been shown that metastatic tumor cells in some tumors can display different biological behaviors due to tumor cell heterogeneity [3]. In the present study, some biological markers in tumor cells from metastatic lymph nodes differed from those of the tumor cells from the primary tumors in CRC cases with lymph node metastases.

NF-*κ*B has an important role in many physiological events such as apoptosis, inflammation, and cell proliferation. Its activation has a regulatory role in expression of more than 400 gene products associated with inflammation, cell survival, proliferation, invasion, and angiogenesis [10–12] Many studies have revealed the role of NF-*κ*B in hematological and solid tumors, previously [10]. The subunits of NF-*κ*B have been shown to have a relation with the development and the progression of solid tumors. Aberrant NF-*κ*B has been shown to be effective through increased proliferation and antiapoptotic mechanisms in colon cancer development as in many other types of cancer. NF-*κ*B is known to be associated with CRC development by increasing inflammation [13]. In the present study, NF-*κ*B expression was higher in the lymph node metastases than in the primary tumors, which is consistent with the results of other previous studies showing that NF-*κ*B plays a role in cancer development and progression [11]. The results of the present study suggest that the cells acquiring the ability to metastasize have more NF-*κ*B expressions in CRC. Increased NF-*κ*B expression in lymph node metastases indicates a potential role of this molecule in the metastasis process of CRC. Although NF-*κ*B is thought to play a role in early stages of CRC development, the idea that it can also be involved in later stages and metastases is supported by the findings of the present study.

Another finding of the present study is that p53 and Ki-67 expressions were significantly higher in the metastatic cells than in the primary tumor cells. As in many other tumors, in CRC, mutations which may occur in the p53 gene may disrupt the activity of NF-*κ*B. In one study, the rate of p53 overexpression was as high as 73% in primary CRC and 66% in distant metastases [25]. The p53 gene alterations are widely considered as a negative prognostic indicator for local control and metastatic spread of the disease [26, 27]. However, conflicting with this idea, findings on no association of p53 expression with either metastatic behavior or tumor aggression are also available [28]. The present study showed that p53 expression was higher in metastatic lymph nodes as compared with the primary tumors. This finding suggests that increased expression of mutant p53 in lymph node metastases, which plays a role in later stages of multistep CRC carcinogenesis, may be linked with increased NF-*κ*B. Actually, the relation between NF-*κ*B and p53 is defined well. Increased NF-*κ*B and p53 expressions in metastatic cells lend support for the idea that these two molecules are multifunctional coregulators. The proliferation marker Ki-67 is utilized as a prognostic indicator, and it is suggested that a biomarker-based approach can be an effective strategy to improve results of adjuvant treatments [6]. In addition, it was reported in a retrospective study that Ki-67 could be closely related to prognosis and overall survival [8]. The results of the present study are also consistent with the view that Ki-67 ratio is a negative prognostic indicator. In the current study, Ki-67 expression in the metastatic lymph nodes was higher, which may be indicative of the aggressive nature of advanced stage tumors. Moreover, the size of the primary tumor was positively correlated with Ki-67.

MMP-1 is frequently found in aggressive tumors. It may not be the basic factor in the initial stage of tumor formation and invasion; however, it plays an effective role in advanced stages of tumors. MMP-1 is the leading enzyme which plays a role in destruction of collagens 1, 2, and 3, the most common stromal structures between the tissues. It has an active role in various tumors such as gastric cancer, breast cancer, squamous cell carcinomas of the head and the neck, colon carcinomas, and adenocarcinomas of pancreas and lungs [20, 22]. In the present study, MMP-1 expression was found to decrease in the metastatic lymph nodes when compared with the primary tumors. The prognostic significance of MMPs in tumor growth and progression has been investigated in a variety of studies [19]. Highly significant differences in the expression of MMP genes (-1, -3, -7, -9, -10, -11, -12, and -14) between primary cancers and their metastases were reported and these findings were associated with aggressive tumor behavior, though the prognostic importance of MMP expression in CRC has not been determined yet [16, 33]. In fact, in the present study, the MMP-1 expression was nonsignificantly decreased in the metastatic lymph nodes. There have been several studies associating NF-*κ*B with MMPs suggesting that migratory genes may support NF-*κ*B in inflammation and carcinogenesis [23, 24, 34]. Considering that NF-*κ*B triggers many extracellular events like tumor angiogenesis, it is very likely to have a relation with MMP-1. In the current study, a positive correlation was not found between MMP-1 and NF-*κ*B expressions, and also MMP-1 expression was decreased nonsignificantly in the metastatic tumors. This may be partly related to the complex stromal structures of metastatic lymph nodes and their microenvironment [20].

The present study also revealed that Ki-67 expression was related to the size of the primary tumor and the number of metastatic lymph nodes. A significant positive correlation was found between NF-*κ*B and both Ki-67 and p53 in the metastatic lymph nodes.

Many potential target molecules have been studied to determine whether they could inhibit NF-*κ*B signal pathway, in cancer cells so far. In the present study, NF-*κ*B, as a transcription factor involved in many physiological and pathological processes, was shown to play a role in CRC progression. It is of particular importance that NF-*κ*B expression was correlated with poor prognostic factors including p53 and Ki-67. At present, many targeted molecules, such as Bortezomib which increases apoptosis by reducing NF-*κ*B levels and decreases resistance to chemotherapy, are under research for the treatment of CRC [35, 36]. In the near future, treatment alternatives directed towards NF-*κ*B, which inhibits nuclear translocation, can be developed. As in the present study, revealing changes in NF-*κ*B in primary and metastatic tumor cells in various types of cancers may accelerate the development of drugs against this molecule.

CRC development is a multifactorial and multistep process. Activation of many oncogenes and inactivation of tumor suppressor genes play roles in this process. Many genetic and epigenetic changes are incorporated in the processes of normal colorectal mucosal epithelial transformation into carcinoma and tumor progression. CRC, with the exception of some well-defined familial syndromes, is mostly sporadic. Approximately 90% of CRC cases are thought to develop from colorectal adenomas with classic adenoma-carcinoma sequences [37]. Changes seen in APC, KRAS, p53, SMAD4, and mis-match repair (MMR) genes are among the most common biological characteristics in CRC carcinogenesis. APC and KRAS are observed in colorectal adenoma and carcinoma with the rate of 40–80% and they play a role in early steps of carcinogenesis [37].

p53 is a molecule in cell replication that arrests the cell cycle when there is DNA damage and when the damage cannot be repaired it leads to apoptosis. As a result of the mutation of p53, cells with damaged DNAs are likely to lead to cancer. Despite thousands of studies in literature, critical mechanisms in which p53 has been involved in carcinogenesis have not yet been fully explained. Mutated p53 has been observed in approximately 50% of all cancer cases and 60% of CRC cases, such that it is called “the guardian of the genome” [38]. Mutated p53 is less frequent in earlier stages of colon adenoma; however, it becomes more frequent in invasive cancers. In CRCs, high levels of mutant p53 are related to high resistance to chemotherapy and worse prognosis [39]. In our study, the p53 expression rates were 91.8% and 97.3% in the primary tumor tissue and the metastatic lymph nodes, respectively. p53 mutations were reported to be 34% in proximal colon tumors and 45% in distal CRCs [40]. In our study, however, p53 positivity was found to be 92.2% in colon tumors and 91.3% in rectal tumors. The results of our study revealing high positivity of p53 in both primary and metastatic tumors support the role of p53 in later clinical stages of CRC.

It is known that an increase in proliferation/apoptosis rate characterizes oncogenesis due to the proliferation of the damaged cells that lose their apoptosis functions as a result of the p53 mutation. In this study, a higher Ki-67 proliferation rate in metastatic lymph nodes than the primary tumor cells may represent the increased mitotic capacity of metastatic cells in CRC.

In addition to playing an important role in inflammation and immune responses, NF-*κ*B is a well-known transcription factor that has a role in development and progression of many cancers. NF-*κ*B is claimed to have a role in apoptosis, angiogenesis, and proliferation effects and development of cancer. Higher expressions of NF-*κ*B in adenomas than that of normal mucosa have been previously stated in colorectal cancer development [38, 41, 42]. In our study, NF-*κ*B, like p53 and Ki-67, has been found to have a higher expression in metastatic lymph nodes than primary tumors in CRC patients.

In CRC, many factors may have a role in tumor progression. According to the results of our study, p53 and NF-*κ*B can have a role in the development of CRC lymph node metastasis. These results showed that tumor cells in CRC lymph node metastasis may display a different biological behavior as compared to that of the primary tumor cells. In CRC patients, adjuvant chemotherapy applications affect the prognosis in lymph node metastases and in view of these results, more aggressive primary tumor cells with increased proliferation and decreased apoptosis rates seem to have caused the lymph node metastases.

In recent years, with the integration of targeted treatments better clinical results are obtained in some selected cancer patients [32]. In our study, except for pT stage, no parameter has been found to have a significant effect on both RFS and 5-year OS rates for the CRC cases treated with classical chemotherapy regimens. It is only possible with a better knowledge of behaviors of metastatic cells that new treatment options can be developed for patients with lymph node metastasis.

In view of the results of this study, it seems that NF-*κ*B activity has a unique role in the course of CRC. Increased NF-*κ*B, p53, and Ki-67 expressions in metastatic tumors may have negative effects on either the natural clinical course or the response to treatments. Unfortunately, the data on microsatellite instability and KRAS mutations are (mostly) not available since this is a retrospective study. These markers would be useful since the presence of such lesions may interact with several pathways such as p53 mutations and also clinical outcomes. The finding that metastatic tumor cells in the lymph nodes have biological features differing from those of the primary tumor cells is particularly important in development of future treatment alternatives, such as NF-*κ*B inhibitors. The results of this study also indicate that in CRC progression many other markers' expressions need to be researched.

## Figures and Tables

**Table 1:** Patients' characteristics (*n* = 110).

Characteristics	*n* (%)
Age (year)	
Range	32–91
Median	58
Sex	
Male	73 (66)
Female	37 (34)
Tumor localization	
Colon	
Right-sided	35 (31.8)
Left-sided	29 (26.4)
Rectum	46 (41.8)
pT category	
pT1	0
pT2	9 (8.2)
pT3	72 (65.5)
pT4	29 (26.3)
Tumor size (cm)	
Range	2.5–12
Mean	5 ± 1.8
Dissected lymph node	
Range	9–48
Mean	18 ± 6
Metastatic lymph node	
Range	1–33
Mean	3 ± 1.2

**Table 2:** NF-*κ*B, MMP-1, p53, and Ki-67 expressions in the primary tumor and the metastatic lymph nodes.

	Colon (*n* = 64)		Rectum (*n* = 46)		All tumors (*n* = 110)	
	Primary tumor	Metastatic lymph node	Primary tumor	Metastatic lymph node	Primary tumor	Metastatic lymph node
NF-*κ*B						
Negative	11 (17.2%)	6 (9.4%)	7 (15.2%)	4 (8.7%)	18 (16.4%)	10 (9.1%)
Weak	6 (9.4%)	5 (7.8%)	6 (13%)	6 (13%)	12 (10.9%)	11 (10%)
Moderate	20 (31.2%)	22 (34.4%)	14 (30.5%)	13 (28.3%)	34 (30.9%)	35 (31.8%)
Strong	27 (42.2%)	31 (48.4%)	19 (41.3%)	23 (50%)	46 (41.8%)	54 (49.1%)
	*P* = 0.04		*P* = 0.04		*P* = 0.04	

MMP-1						
Negative	12 (18.8%)	21 (32.8%)	8 (17.4%)	15 (32.6%)	20 (18.2%)	36 (32.7%)
Weak	29 (45.3%)	16 (25%)	21 (45.7%)	11 (23.9%)	50 (45.5%)	27 (24.6%)
Moderate	14 (21.8%)	25 (39.1%)	11 (23.9%)	19 (41.3%)	25 (22.7%)	44 (40%)
Strong	9 (14.1%)	2 (3.1%)	6 (13%)	1 (2.2%)	15 (13.6%)	3 (2.7%)
	*P* = 0.08		*P* = 0.07		*P* = 0.07	

p53						
Negative	5 (7.8%)	2 (3.1%)	4 (8.7%)	1 (2.2%)	9 (8.2%)	3 (2.7%)
Weak	11 (17.2%)	6 (9.4%)	13 (28.3%)	4 (8.7%)	24 (21.8%)	10 (9.1%)
Moderate	31 (48.4%)	36 (56.3%)	19 (41.3%)	23 (50%)	50 (45.5%)	59 (53.6%)
Strong	17 (26.6%)	20 (31.2%)	10 (21.7%)	18 (39.1%)	27 (24.5%)	38 (34.6%)
	*P* = 0.04		*P* = 0.03		*P* = 0.04	

Ki-67						
Negative	0 (0%)	0 (0%)	0 (0%)	0 (0%)	0 (0%)	0 (0%)
Weak	32 (50%)	13 (20.3%)	20 (43.5%)	8 (17.4%)	52 (47.3%)	21 (19.1%)
Moderate	21 (32.8%)	31 (48.4%)	15 (32.6%)	18 (39.1%)	36 (32.7%)	49 (44.5%)
Strong	11 (17.2%)	20 (31.3%)	11 (23.9%)	20 (43.5%)	22 (20%)	40 (36.4%)
	*P* = 0.01		*P* = 0.02		*P* = 0.01	

## References

[1] Jemal A., Thomas A., Murray T., Thun M. (2002). Cancer statistics, 2002. *Ca—A Cancer Journal for Clinicians*.

[2] Meteoglu I., Meydan N., Erkus M. (2008). Id-1: regulator of EGFR and VEGF and potential target for colorectal cancer therapy. *Journal of Experimental and Clinical Cancer Research*.

[3] Dikicioglu E., Barutca S., Meydan N., Meteoglu I. (2005). Biological characteristics of breast cancer at the primary tumour and the involved lymph nodes. *International Journal of Clinical Practice*.

[4] Cohen A. M., Tremiterra S., Candela F., Thaler H. T., Sigurdson E. R. (1991). Prognosis of node-positive colon cancer. *Cancer*.

[5] Hamilton S. R. (2008). Targeted therapy of cancer: new roles for pathologists in colorectal cancer. *Modern Pathology*.

[6] Graziano F., Cascinu S. (2003). Prognostic molecular markers for planning adjuvant chemotherapy trials in Dukes' B colorectal cancer patients: how much evidence is enough?. *Annals of Oncology*.

[7] Tanigawa N., Amaya H., Matsumura M. (1997). Tumor anglogenesis and mode of metastasis in patients with colorectal cancer. *Cancer Research*.

[8] Garrity M. M., Burgart L. J., Mahoney M. R. (2004). Prognostic value of proliferation, apoptosis, defective DNA mismatch repair, and p53 overexpression in patients with resected Dukes' B2 or C colon cancer: a north central cancer treatment group study. *Journal of Clinical Oncology*.

[9] Giatromanolaki A., Stathopoulos G. P., Tsiobanou E. (1999). Combined role of tumor angiogenesis, bcl-2, and p53 expression in the prognosis of patients with colorectal carcinoma. *Cancer*.

[10] Meteoglu I., Erdogdu I. H., Meydan N., Erkus M., Barutca S. (2008). NF-kappaB expression correlates with apoptosis and angiogenesis in clear cell renal cell carcinoma tissues. *Journal of Experimental and Clinical Cancer Research*.

[11] Vaiopoulos A. G., Athanasoula K. C., Papavassiliou A. G. (2013). NF-*κ*B in colorectal cancer. *Journal of Molecular Medicine*.

[12] Hassanzadeh P. (2011). Colorectal cancer and NF-*κ*B signaling pathway. *Gastroenterology and Hepatology from Bed to Bench*.

[13] Abdullah M., Rani A. A., Sudoyo A. W., Makmun D., Handjari D. R., Hernowo B. S. (2013). Expression of NF-kB and COX2 in colorectal cancer among native Indonesians: the role of inflammation in colorectal carcinogenesis. *Acta Medica Indonesiana*.

[14] Vaiopoulos A. G., Papachroni K. K., Papavassiliou A. G. (2010). Colon carcinogenesis: Learning from NF-kappaB and AP-1. *International Journal of Biochemistry and Cell Biology*.

[15] Zucker S., Vacirca J. (2004). Role of matrix metalloproteinases (MMPs) in colorectal cancer. *Cancer and Metastasis Reviews*.

[16] Asano T., Tada M., Cheng S. (2008). Prognostic values of matrix metalloproteinase family expression in human colorectal carcinoma. *Journal of Surgical Research*.

[17] Gill S. E., Parks W. C. (2008). Metalloproteinases and their inhibitors: regulators of wound healing. *International Journal of Biochemistry and Cell Biology*.

[18] Overall C. M., Kleifeld O. (2006). Validating matrix metalloproteinases as drug targets and anti-targets for cancer therapy. *Nature Reviews Cancer*.

[19] Shiozawa J., Ito M., Nakayama T., Nakashima M., Kohno S., Sekine I. (2000). Expression of matrix metalloproteinase-1 in human colorectal carcinoma. *Modern Pathology*.

[20] Noël A., Jost M., Maquoi E. (2008). Matrix metalloproteinases at cancer tumor-host interface. *Seminars in Cell and Developmental Biology*.

[21] Møller Sørensen N., Vejgaard Sørensen I., Ørnbjerg Wurtz S. (2008). Biology and potential clinical implications of tissue inhibitor of metalloproteinases-1 in colorectal cancer treatment. *Scandinavian Journal of Gastroenterology*.

[22] Hinoda Y., Okayama N., Takano N. (2002). Association of functional polymorphisms of matrix metalloproteinase (MMP)-1 and MMP-3 genes with colorectal cancer. *International Journal of Cancer*.

[23] Wu Y., Zhang X., Zhou H. (2013). Factor VIIa regulates the expression of caspase-3, MMP-9, and CD44 in SW620 colon cancer cells involving PAR2/MAPKs/NF-*κ*B signaling pathways. *Cancer Investigation*.

[24] Yeh C.-B., Hsieh M.-J., Hsieh Y.-H., Chien M.-H., Chiou H.-L., Yang S.-F. (2012). Antimetastatic effects of norcantharidin on hepatocellular carcinoma by transcriptional inhibition of MMP-9 through modulation of NF-Kb activity. *PLoS ONE*.

[25] Menon A. G., Tollenaar R. A. E. M., van de Velde C. J. H. (2004). p53 and HLA class-I expression are not down-regulated in colorectal cancer liver metastases. *Clinical & Experimental Metastasis*.

[26] Ito T., Kaneko K., Makino R. (2003). Clinical significance in molecular detection of p53 mutation in serum of patients with colorectal carcinoma. *Oncology Reports*.

[27] Escandell J. M., Kaler P., Recio M. C. (2008). Activated K-ras protects colon cancer cells from cucurbitacin-induced apoptosis: the role of p53 and p21. *Biochemical Pharmacology*.

[28] Lebe B., Sarioglu S., Sokmen S., Ellidokuz H., Fuzun M., Kupelioglu A. (2005). The clinical significance of p53, p21, and p27 expressions in rectal carcinoma. *Applied Immunohistochemistry & Molecular Morphology*.

[29] de Jong K. P., Gouw A. S. H., Peeters P. M. J. G. (2005). P53 mutation analysis of colorectal liver metastases: relation to actual survival, angiogenic status, and p53 overexpression. *Clinical Cancer Research*.

[30] Zavrides H., Zizi-Sermpetzoglou A., Elemenoglou I. (2006). Immunohistochemical expression of bcl-2 in UICC stage I and III colorectal carcinoma patients: correlation with c-erbB-2, p53, ki-67, CD44, laminin and collagen IV in evaluating prognostic significance. *Polish Journal of Pathology*.

[31] Zhigang Z., Wenlv S. (2004). Prostate Stem Cell Antigen (PSCA) expression in human prostate cancer tissues: implications for prostate carcinogenesis and progression of prostate cancer. *Japanese Journal of Clinical Oncology*.

[32] Akkad J., Bochum S., Martens U. M. (2015). Personalized treatment for colorectal cancer: novel developments and putative therapeutic strategies. *Langenbeck's Archives of Surgery*.

[33] Baker E. A., Bergin F. G., Leaper D. J. (2000). Matrix metalloproteinases, their tissue inhibitors and colorectal cancer staging. *British Journal of Surgery*.

[34] Yang C., Yan J., Yuan G. (2014). Icotinib inhibits the invasion of Tca8113 cells via downregulation of nuclear factor *κ*B-mediated matrix metalloproteinase expression. *Oncology Letters*.

[35] Voutsadakis I. A., Patrikidou A., Tsapakidis K. (2010). Additive inhibition of colorectal cancer cell lines by aspirin and bortezomib. *International Journal of Colorectal Disease*.

[36] O'Neil B. H., Raftery L., Calvo B. F. (2010). A phase i study of bortezomib in combination with standard 5-fluorouracil and external-beam radiation therapy for the treatment of locally advanced or metastatic rectal cancer. *Clinical Colorectal Cancer*.

[37] Arends M. J. (2013). Pathways of colorectal carcinogenesis. *Applied Immunohistochemistry and Molecular Morphology*.

[38] Xiao-Lan L., Zhou J., Chen Z. R., Chng W. J. (2015). P53 mutations in colorectal cancer—molecular pathogenesis and pharmacological reactivation. *World Journal of Gastroenterology*.

[39] Allegra C. J., Paik S., Colangelo L. H. (2003). Prognostic value of thymidylate synthase, Ki-67, and p53 in patients with Dukes' B and C colon cancer: a National Cancer Institute-National Surgical Adjuvant Breast and Bowel Project collaborative study. *Journal of Clinical Oncology*.

[40] Noske A., Lipka S., Budczies J. (2009). Combination of p53 expression and p21 loss has an independent prognostic impact on sporadic colorectal cancer. *Oncology Reports*.

[41] Hassanzadeh P. (2011). Colorectal cancer and NF-*κ*B signaling pathway. *Gastroenterology and Hepatology*.

[42] Aranha M. M., Borralho P. M., Ravasco P. (2007). NF-*κ*B and apoptosis in colorectal tumourigenesis. *European Journal of Clinical Investigation*.

